# Simultaneous point‐of‐care testing of blood lipid profile and glucose: Performance evaluation of the GCare Lipid Analyzer

**DOI:** 10.1002/jcla.24055

**Published:** 2021-10-25

**Authors:** Ha Nui Kim, Soo‐Young Yoon

**Affiliations:** ^1^ Department of Laboratory Medicine Korea University Guro Hospital Seoul Korea

**Keywords:** diabetes mellitus, dyslipidemia, glucose, lipid, POC, point‐of‐care testing

## Abstract

**Background:**

Point‐of‐care (POC) testing provides quick results and includes tests for blood glucose and lipid profiles. We evaluated the newly developed POC device, the GCare Lipid Analyzer, which is used to measure glucose, total cholesterol (TC), triglyceride (TG), and high‐density lipoprotein cholesterol (HDL‐C) levels.

**Methods:**

Venous and capillary blood samples were collected from patients who visited Korea University Guro Hospital. The results obtained using the GCare Lipid Analyzer were compared with those obtained using the TBA 2000FR chemistry analyzer and YSI 2300 STAT Plus analyzer. The glucose system evaluation process was based on the International Organization for Standardization 15197:2013 guidelines.

**Results:**

The correlation coefficients (R) for TC, TG, and HDL‐C were 0.965, 0.969, and 0.943 in capillary blood and 0.969, 0.990, and 0.956 in venous blood, respectively. The total errors for TC, TG, and HDL‐C of the lipid profile using venous blood were all acceptable at 6.6%, 9.3%, and 11.6%, respectively. For glucose concentrations <100 mg/dl, 96.1% of the measured glucose levels were within ±15 mg/dl in venous samples and 100% were within ±15 mg/dl in capillary samples. For glucose concentrations ≥100 mg/dl, 100% and 99.5% of the measured glucose levels were within 15% for venous and capillary blood, respectively.

**Conclusion:**

The performance of the GCare Lipid Analyzer is acceptable for both blood glucose and lipid profile testing, indicating that it is reliable for use in patients with diabetic dyslipidemia.

## Graphical abstract

This study aims to introduce a newly developed POC device, the GCare Lipid Analyzer, which is used to measure glucose, total cholesterol (TC), triglyceride (TG), and high‐density lipoprotein cholesterol (HDL‐C) levels. We found that the performance of the GCare Lipid Analyzer is acceptable for both blood glucose and lipid profile testing, indicating that it is reliable for use in patients with diabetic dyslipidemia.

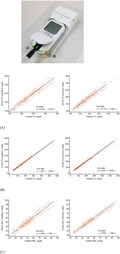

## 1 INTRODUCTION

Dyslipidemia, which poses a significant threat to the public health system, is on the rise worldwide, with a reported prevalence of 42.7% and 56.8% in China and the United States, respectively.^1^ Serum lipids are strongly affected by insulin; thus, dyslipidemia is a common feature of diabetes mellitus (DM).^2^ The coexistence of DM and dyslipidemia is termed diabetic dyslipidemia and is common in individuals with type 2 DM.^3^ Dyslipidemia is also known to be involved in the development of various diseases, such as chronic kidney disease, metabolic syndrome, obesity, hypertension, and cardiovascular disease (CVD) and associated with nutrient supplementation, such as magnesium.^1^, ^4^, ^5^, ^6^ The lipoprotein pattern observed in patients with diabetic dyslipidemia includes elevated fasting and postprandial triglycerides (TG), low high‐density lipoprotein (HDL‐C), elevated low‐density lipoprotein (LDL‐C), and the predominance of small dense LDL particles.^7^

The decision to start treatment for dyslipidemia is based on the analysis of lipid fractions including TC, HDL‐C, LDL‐C, and non‐HDL‐C.^8^ This baseline lipid evaluation is performed periodically after initiating pharmacological interventions such as statins. Patients are required to visit the hospital laboratory to provide blood samples for lipid profile testing before their outpatient clinic visit. The use of point‐of‐care (POC) devices in this situation is expected to alleviate this inconvenience. Several hand‐held portable POC devices are currently available to measure lipid and glucose levels in the blood, such as the Accutrend Plus, Bene Check Plus, CardioChek PA, Veri‐Q, 3 in 1, and elemark™, as well as the compact desktop analyzer, the Cholestech LDX^®^. These devices can measure lipid profiles and ratios in whole blood, plasma, or serum using reflectance or biosensor technology and feature disposable strips, rotors, or cassettes.^9^ However, most patients with diabetes are unfamiliar with systems for self‐monitoring lipid profiles, unlike commonly used self‐monitoring blood glucose devices.

Here, we present a new laboratory information system developed in South Korea, a connectable hand‐held glucose and lipid (TC, TG, HDL‐C, calculated LDL‐C) monitoring system named the GCare Lipid Analyzer (Green Cross Medical Science, Yongin, Korea). Considering that this is the first study on the performance of this POC device, we evaluated the glucometer's performance in accordance with the International Organization for Standardization (ISO) 15197:2013 guidelines and assessed lipid measurements for precision, accuracy, and correlation with values obtained using a TBA2000FR chemistry analyzer.

## 2 MATERIAL AND METHODS

### 2.1 Subjects

We recruited two groups of adult volunteers (age range, 19–80 years) among patients who visited Korea University Guro Hospital for their prescribed blood tests. For the evaluation of the lipid panel, an additional 2 ml EDTA venous blood and 150 *µ*L of capillary blood were collected from the study subjects (*n* = 136). Specimens in which the hematocrit (Hct) range did not fall within 25%–60% were excluded. An additional 10 ml of venous blood (into an EDTA tube) and 150.5 µl of capillary blood were collected from the group (*n* = 100) and tested for blood glucose concentration, complete blood count, and blood type. Specimens that did not meet the Hct range of 15%–65% were excluded from the evaluation.

### 2.2 GCare Lipid Analyzer

**FIGURE 1:**
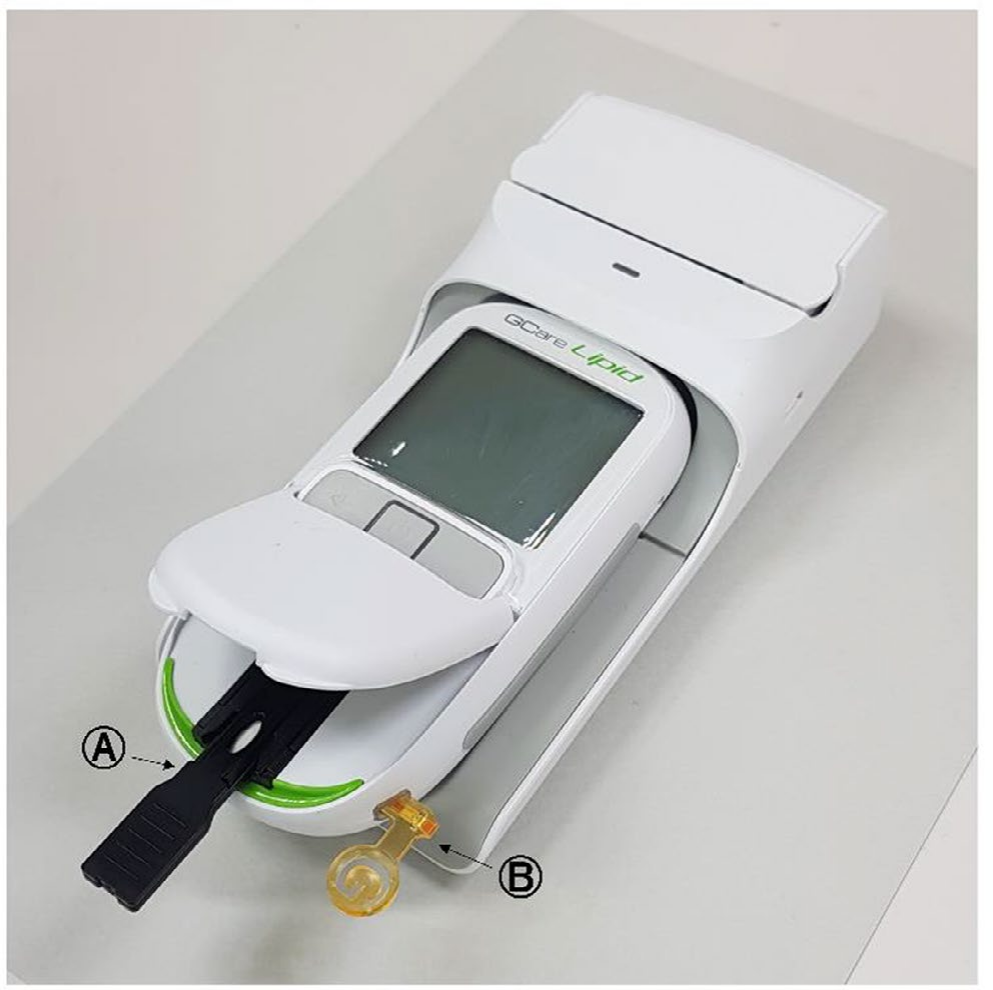
Picture of the GCare Lipid Analyzer showing its two inlets, (A) one for lipid panel testing using a GCare Lipid Profile Test Strip; and (B) another for glucose testing using a GCare Glucose Test Strip. Courtesy: Green Cross Medical Science

GCare Lipid Analyzer (Green Cross Medical Science) is a compact, hand‐held combined lipid and glucose POC device. Both capillary and venous blood samples are available for testing. The device is similar to most other blood glucometers, except that it features two slots that enable users to easily select tests according to their needs. The inlet at the bottom accommodates the lipid test strip, and the smaller inlet on the left side is for the blood glucose test strip (Figure 1). The GCare Lipid Profile Test Strip was used, which incorporated an enzymatic colorimetric method, to measure TC, TG, and HDL‐C. The LDL‐C value is calculated from the TC, TG, and HDL‐C values measured by the device, according to the Friedewald formula (TC ‐ HDL‐C ‐ TG/5) when the TG value is <350 mg/dl. The measurement time for the lipid profiles is 180 s, and the required sample volume is 40 µl. Each measured lipid parameter can be checked on the screen of the device by clicking the arrow button on the device serially. For the glucose level testing, the GCare Glucose Test Strip is used which is based on the glucose dehydrogenase flavin adenine dinucleotide system. The sample volume required for measurement is 0.5 µl and the measuring time is 5 s.

### 2.3 Study design for the GCare Lipid Analyzer using GCare Lipid Profile Test Strip

For the precision evaluation of the lipid panel (TC, TG, and HDL‐C), three concentrations (low, medium, and high) of venous blood were measured 10 times using three lots according to the Clinical and Laboratory Standards Institute (CLSI) EP5‐A3.^10^ Analytical precision was assessed by calculating each measured value per lot as mean, standard deviation (SD), and coefficient of variation (CV, %). Standardization is important for the measurement of lipoproteins because treatment decision points have been established by the expert consensus of the National Cholesterol Education Program (NCEP).^11^ The NCEP states that the analytical performance criteria in terms of total error (%) for TC, TG, and HDL‐C are ≤8.9%, ≤15%, and ≤13%, respectively, while the certification criteria for imprecision (CV, %) of TC, TG, and HDL‐C are ≤3%, ≤5%, and ≤4%, respectively.^12^, ^13^, ^14^, ^15^ Regarding the standardization criteria of the Centers for Disease Control (CDC) for lipids and lipoproteins, bias, and imprecision are considered separately, although the values of acceptable bias (%) and CV are the same as those prescribed by the NCEP.

The measurement procedure comparison study was conducted according to CLSI document EP9.^16^ Each measurement was tested in duplicate and was compared with the reference value obtained from the TBA2000FR chemistry analyzer (Toshiba Co., Ltd.) using plasma samples from whole blood in EDTA tubes. Determiner C‐TC, Determiner‐C TG, and Determiner‐L‐HDL‐C (Kyowa Medex Co., Ltd.) were used as reagents for measuring TC, TG, and HDL‐C on the TBA2000FR, respectively. Based on the Adult Treatment Panel (ATP) III risk guideline,^17^ the samples were subdivided into three categories according to their test results on the TBA2000FR. The standard levels per the ATP III guideline are as follows: (1) TG (mg/dl) <200, 200–239, and ≥240; (2) TG (mg/dl) <150, 150–199, and ≥200; and (3) HDL‐C (mg/dl) <40, 40–60, and ≥60. These criteria were also applied to the analysis of clinical agreement. The mean bias of TC, TG, and HDL‐C was calculated as a percentage: (GCare – Toshiba)/Toshiba ×100. The Bland–Altman analysis and Passing–Bablok regression analysis were performed to determine method agreement.

For the user performance evaluation, the recruited volunteers read the device manual and collected their capillary blood on their own, without the help of the technicians. When the measurement was completed by the user, the technician immediately collected the capillary blood of the users and measured it again using the same device. The values obtained by the user and technician were then compared.

### 2.4 Study design for the GCare Lipid Analyzer using GCare Glucose Test Strip

The blood glucose meter evaluations were performed in accordance with the ISO15197:2013 guidelines.^18^ Tests to evaluate precision, the effect of Hct and interfering substances, and accuracy, including the user performance evaluation, were conducted. The precision evaluation was conducted using 10 meters, three lots, and five samples with different glucose concentrations ranging from low to high concentrations. Similarly, five different Hct levels (20%, 30%, 40%, 50%, and 60%) and three lots at three glucose concentrations specified in the ISO 15197:2013 guidelines were used to evaluate the Hct. Hct levels were measured using the HemoCue^®^ Hb 301 System (HemoCue AB, Ängelholm, Sweden). Each measurement was taken 10 times with two devices and three lots to obtain a total of 60 results. The acceptance criteria for the difference between the average concentration of glucose measured at each Hct level and the average concentration measured at the mid‐level Hct level were within ±10 mg/dl for glucose levels ≤100 mg/dl and within ±10% for glucose levels >100 mg/dl. The influences of 24 possible interfering substances were evaluated using two glucose concentrations and three lots. The acceptable difference between the mean blood glucose level of the normal samples and that of the samples containing interfering substances was within ±10 mg/dl for glucose levels ≤100 mg/dl and within ±10% for glucose levels >100 mg/dl. For accuracy testing, each 100 venous and capillary blood samples were tested in duplicate using each of three different reagent lots. The ISO guidelines were referred to obtain the stipulated minimum system accuracy performance criteria for glucometers. Among the measured glucose values, >95% should be within ±15 mg/dl of the average measured values of the reference measurement at glucose concentrations <100 mg/dl or within ±15% at glucose concentrations >100 mg/dl. Further, 99% of the measured values should fall within zones A and B of the consensus error grid (CEG). The reference value was obtained using the YSI 2300 Plus STAT analyzer (YSI Inc), the most widely used device for determining the accuracy of blood glucose measurement. User performance was evaluated using the capillary samples of 100 volunteers with diabetes, utilizing one reagent lot under the supervision of a healthcare provider; the device use instructions were supplied.

### 2.5 Statistical analysis

Data recording and processing were performed using Microsoft Excel 2016. The correlation analysis and graph generation were assessed by Bland–Altman and Passing–Bablok regression analyses using MedCalc^®^ Statistical Software version 19.8 (MedCalc Software Ltd).

### 2.6 Ethical approval

This study was approved by the Institutional Review Board of Korea University Guro Hospital (2019GR0206 for glucose; 2019GR0365 for lipids). All enrolled participants who met the inclusion criteria for the study provided their informed consent.

## 3 RESULTS

### 3.1 Precision profile of the GCare Lipid Analyzer

The precision profiles of the GCare Lipid Analyzer are presented in Table S1. The NCEP‐recommended performance criteria for precision were met in three lots and at low to high TG concentrations (≤5%). TC showed an acceptable CV (≤3%) only at high concentrations in lots 1 and 3, while other CV values did not exceed 5%. The HDL‐C measurements meet the performance criteria (CV ≤4%) at low and high level in lot 2 and at high level in lot 3, while lot 1 failed to meet the criteria in all levels.

The grand average, pooled variance, pooled SD, and pooled CV for each glucose concentration were calculated using the results from all three reagent lots. Pooled SD for levels 1 and 2 were 2.9 mg/dl and 3.0 mg/dl, respectively. Pooled CV values for levels 3, 4, and 5 were 3.0%, 2.6%, and 1.9%, respectively.

### 3.2 Comparison of measurements of the GCare Lipid Analyzer and laboratory measurements including user performance evaluation and sample type comparison

**FIGURE 2:**
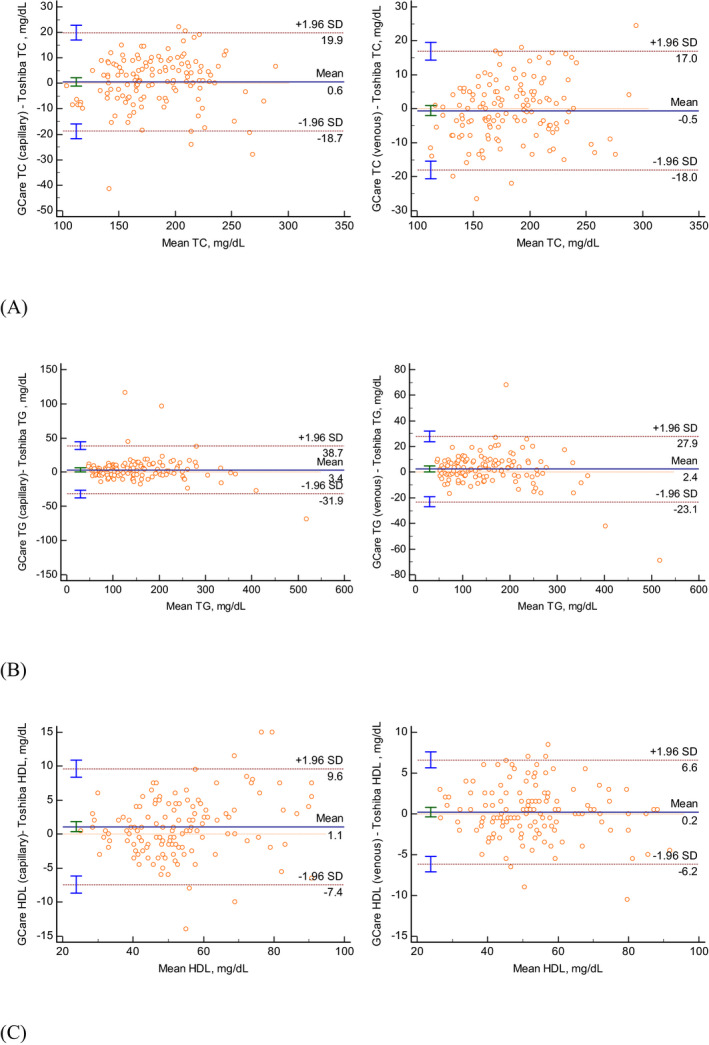
Bland–Altman plot of the data obtained using the GCare Lipid Analyzer for: (A) total cholesterol (TC), (B) triglycerides (TG), and (C) high‐density lipoprotein cholesterol (HDL‐C) in capillary and venous blood samples, compared to the mean TC, TG, and HDL‐C using the reference values obtained using the Toshiba TBA TBA2000FR chemistry analyzer

The measurements of TC, TG, and HDL‐C in the GCare Lipid Analyzer were compared with those of the Toshiba laboratory method as a reference. Bias was estimated for the GCare Lipid Analyzer and the laboratory method using paired results. The mean bias values for TC, TG, and HDL‐C were 0.560, 3.391, and 1.121, respectively, in capillary blood and −0.527, 2.385, and 0.221 in venous blood, respectively. The greatest bias was observed for TG in both types of specimens. The Bland–Altman plots for each lipid test in capillary and venous blood, including the 95% limit of agreement, are displayed in Figure 2.

**FIGURE 3:**
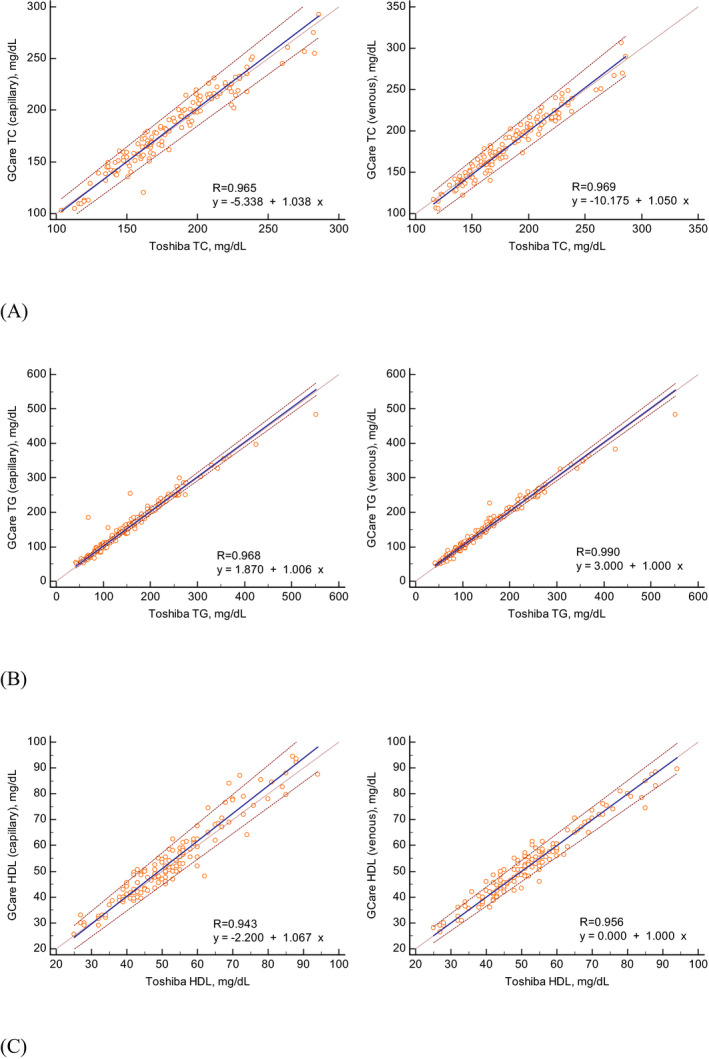
Passing–Bablok regression analysis of the GCare Lipid Analyzer findings for capillary and venous blood samples compared to the reference levels estimated by the Toshiba TBA2000FR chemistry analyzer for (A) total cholesterol (TC), (B) triglyceride (TG), and (C) high‐density lipoprotein cholesterol (HDL‐C)

The Passing–Bablok regression analysis revealed a good to excellent correlation between the GCare Lipid Analyzer and the laboratory method (Figure 3, Table S2). The correlation coefficients (R) for TC, TG, and HDL‐C were 0.965, 0.968, and 0.943 in capillary blood, respectively, while the R values for venous blood were 0.969, 0.990, and 0.956, respectively. All of the R values were >0.950, reflecting excellent correlations, except that for HDL‐C in the capillary blood specimen, which was 0.943.

The correlation between the results of the capillary blood from the user and those acquired through the laboratory method was acceptable at R = 0.957 for TC, 0.991 for TG, and 0.936 for HDL‐C. The mean bias was also within the criteria suggested in the NCEP guidelines: −2.1% for TC, 1.5% for TG, and 3.6% for HDL‐C. In addition, a comparison of the results obtained by the user and the technician revealed acceptable correlations and biases (Table 1). In both evaluations, HDL‐C had the lowest R and highest bias.

**TABLE 1:** Correlation analysis and mean bias (%) of user performance, comparison with technician‐derived values, and comparison of sample types (capillary and venous blood samples) using the GCare Lipid Analyzer

	Lipid test	Correlation coefficient (R)	Bias (%)
User performance	TC	0.957 (0.938–0.971)	−2.1
	TG	0.991 (0.986–0.994)	1.5
	HDL‐C	0.936 (0.908–0.955)	3.6
User vs. technician	TC	0.963 (0.947–0.975)	2.0
	TG	0.993 (0.989–0.995)	0.6
	HDL‐C	0.939 (0.912–0.958)	−2.4
Sample types	TC	0.945 (0.928–0.957)	−0.7
	TG	0.988 (0.984–0.991)	0.0
	HDL‐C	0.926 (0.904–0.942)	−1.8

Abbreviations: HDL‐C, high‐density lipoprotein cholesterol; TC, total cholesterol; TG, triglyceride.

The comparison of sample types revealed good correlations for TC (R = 0.945), TG (R = 0.988), and HDL‐C (R = 0.926). The mean bias was −0.7% for TC, 0.0% for TG, and −1.8% for HDL‐C.

### 3.3 Analytical performance of GCare Lipid Analyzer according to NCEP recommendations

Combining the lowest to highest CV as the overall analytical imprecision (CVa, %) and the mean bias calculated above, the total error was calculated for each test analyte (Table 2). The total error for the venous blood specimens was calculated as bias +1.96 × CVa.^19^ The total errors for TC, TG, and HDL‐C of the lipid system using venous blood were all acceptable at 6.6%, 9.3%, and 11.6%, respectively. The overall analytical imprecision for TC and HDL‐C exceeded 3% and 4%, respectively.

**TABLE 2:** NCEP criteria for analytical performance of lipid and lipoprotein measurements compared with those of the GCare Lipid Analyzer

Test	NCEP performance criteria			GCare Lipid Analyzer (venous blood)		
	Total error (%)	Bias (%)	Imprecisio*n* (CV, %)	Total error (%)	Bias (%)	Overall analytical imprecisio*n* (CV, %)
TC	≤8.9	≤±3	≤ 3	6.6	−0.4	3.6
TG	≤15	≤±5	≤ 5	9.3	1.9	3.8
HDL‐C	≤13	≤±5	≤ 4	11.6	0.8	5.5

Abbreviations: CV, coefficient of variation; HDL‐C, high‐density lipoprotein cholesterol; NCEP, National Cholesterol Education Program; TC, total cholesterol; TG, triglyceride.

### 3.4 Clinical agreement according to ATP III risk categories

Each result measured by the GCare Lipid Analyzer was categorized based on the ATP III guidelines. Whether the category to which the value of GCare Lipid Analyzer belonged to the category to which the reference Toshiba value belonged was calculated as a percentage. “Agreement” was defined as the two results being in the same category, while “disagreement” was not in the same category, such as a difference of one or more category. The categorized results for the capillary and venous blood samples are displayed in Table 3. The agreement (%) was >90% for all categories. All cases of disagreement occurred in only one category, belonging to those below or above.

**TABLE 3:** Clinical agreement according to the adult treatment panel III risk categories between the Toshiba TBA2000FR and GCare Lipid Analyzer

Test	Total number	Capillary blood		Venous blood	
		Agreement (*n*, %)	Disagreement (*n*, %)	Agreement (*n*, %)	Disagreement (*n*, %)
TC (mg/dl)					
Total	220	203 (92.3%)	17 (7.7%)	201 (91.4%)	19 (8.6%)
<200	156		11 (7.1%)		10 (6.4%)
≥200 and <240	56		6 (10.7%)		9 (16.1%)
≥240	8		0 (0.0%)		0 (0.0%)
TG (mg/dl)					
Total	200	183 (91.5%)	17 (8.5%)	191 (95.5%)	9 (4.5%)
<150	110		6 (5.5%)		3 (2.7%)
≥150 and <200	36		10 (27.8%)		6 (16.7%)
≥200	54		**1 (1.9%)**		0 (0.0%)
HDL‐C (mg/dl)					
Total	224	209 (93.3%)	15 (6.7%)	208 (92.9%)	16 (7.1%)
<40	34		9 (26.5%)		7 (20.6%)
>40 and <60	146		6 (4.1%)		9 (4.7%)
≥60	44		0 (0.0%)		0 (0.0%)

Abbreviations: HDL‐C, high‐density lipoprotein cholesterol; TC, total cholesterol; TG, triglycerides.

The greatest disagreement in venous blood was observed for TC (8.6%). All misclassified cases with a TC <200 mg/dl had slightly overestimated TC levels; thus, they belonged to the upper categories of ≥200 mg/dl and <240 mg/dl. In the 10 overestimated cases, TC levels ranged from 200 to 210 mg/dl (mean value: 203.8 mg/dl), including four cases with a reference cut‐off value of 200 mg/dl. The disagreement rate for TG in the capillary blood (8.5%) was greater than that in the venous blood (4.5%). Among the 10 discordant cases of capillary blood in the TG level range of ≥150 to <200 mg/dl, six were misclassified as <150 mg/dl and four were in the ≥200 mg/dl group, revealing no significant systematic trend. In HDL‐C, 20.6%–26.5% of cases with a level <40 mg/dl were falsely higher and misclassified into the above categories (>40 and >60 mg/dl).

### 3.5 Evaluation of interfering substances in the GCare Lipid Analyzer using the GCare Glucose Test Strip

Potential substances that can influence glucose levels, including Hct, were evaluated. The difference between the average measured value at each Hct level and the average measured value at the mid‐level Hct for glucose concentrations <100 mg/dl was less than ±10.0 mg/dl, and less than ±10.0% for glucose concentrations >100 mg/dl (Table S3). To test the effect of interfering substances, a dose‐response evaluation was performed of the six interfering substances (dopamine, gentisic acid, glutathione, urate, methyldopa, and tolazamide) because the results were affected by interference at the tested concentrations. The concentration of each interfering substance not affecting the glucose measurements is summarized in Table S4.

### 3.6 Accuracy of the GCare Lipid Analyzer using the GCare glucose test strip

**FIGURE 4:**
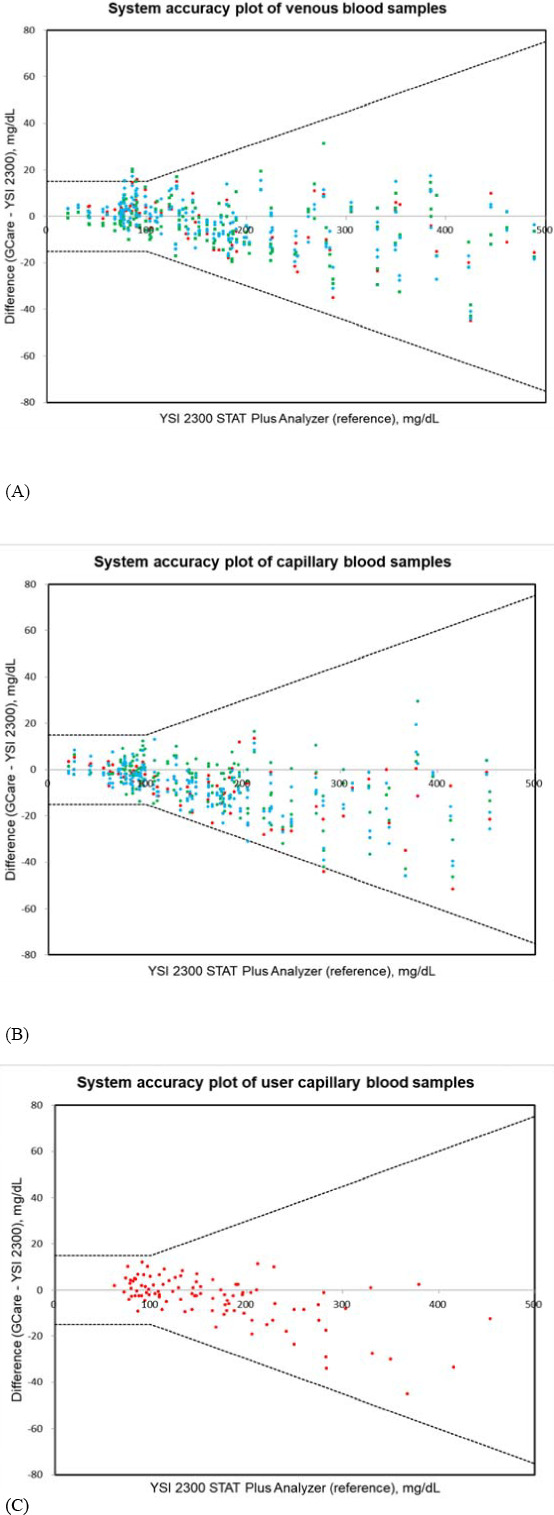
System accuracy plot of the measured blood glucose levels using the GCare Lipid Analyzer with a GCare Glucose Test Strip compared with those obtained using the YSI 2300 STAT Plus Analyzer (reference value), obtained from three different lots using (A) venous and (B) capillary blood samples. (C) Plot of user capillary blood glucose results obtained from one lot

The capillary and venous specimens were distributed according to the concentration intervals specified in the ISO 15197:2013 guidelines (Table S5). Some modified (spiked) samples were included in very low and very high glucose concentrations, as indicated by the numbers in parentheses. The system accuracy results for venous and capillary blood specimens are summarized in Table 4 and Figure 4. For glucose concentrations <100 mg/dl, 96.1% and 100% of the measured glucose levels were within ±15 mg/dl for the venous and capillary blood samples, respectively. For glucose concentrations ≥100 mg/dl, 100% and 99.5% of the measured glucose levels were within 15% for the venous and capillary blood samples, respectively. The CEG analysis (Figure S1) revealed that all measured glucose levels were located within Zones A and B for the venous and capillary blood specimens.

**TABLE 4:** System accuracy results of the GCare Lipid Analyzer using GCare glucose test strip according to ISO 15197:2013 guidelines

Specimen type	Glucose concentrations <100 mg/dl			Glucose concentrations ≥100 mg/dl			Glucose concentrations between 21 mg/dl and 488 mg/dl (venous) 20 mg/dl and 505 mg/dl (capillary) 62 mg/dl and 453 mg/dl (user)
	Within ±5 mg/dl	Within ±10 mg/dl	Within ±15 mg/dl	Within ±5%	Within ±10%	Within ±15%	Within ±15 mg/dl or ±15%
Venous	69.1% (141/204)	90.2% (184/204)	96.1% (196/204)	64.4% (255/396)	96.2% (381/396)	100% (396/396)	98.7% (592/600)
Capillary	66.2% (143/216)	96.8% (209/216)	100% (216/216)	52.6% (202/384)	87.8% (337/384)	99.5% (382/384)	99.7% (598/600)
User (capillary)	71.4% (20/28)	89.3% (25/28)	100% (28/28)	65.3% (47/72)	95.8% (69/72)	100% (73/73)	100% (200/200)

Abbreviation: ISO, International Organization for Standardization.

### 3.7 User performance evaluation of the GCare Lipid Analyzer using GCare glucose test strip

A total of 100 volunteers with diabetes representing different ages, genders, and education levels were recruited to evaluate the GCare Lipid Analyzer using GCare Glucose Test Strip (Table S6). The blood glucose levels in the capillary blood measured by the test subjects were compared to the reference values obtained using the YSI 2300 STAT Plus analyzer. All glucose levels measured by the users were within ±15 mg/dl and ±15% when compared with the reference values (Table 4, Figure 4C). The CEG analysis demonstrated that all the results (100%) obtained by the users from capillary blood were within Zone A (Figure S1C).

## 4 DISCUSSION

The lipid or lipoprotein test results form the basis for treatment policies in guidelines of dyslipidemia. According to the 2019 European guideline,^20^ the treatment of hypertriglyceridemia is recommended at values of TG >200 mg/dl and HDL‐C <40 mg/dl in patients at high cardiovascular risk. The therapeutic goal of LDL‐C is subdivided according to risk status of each patients: <100 mg/dl for those at moderate risk, <70 mg/dl for those at high cardiovascular risk, <55 mg/dl for those at very high cardiovascular risk, and even lower, <40 mg/dl, for those who had a second vascular event during treatment of maximum dose of statins. As several meta‐analyses revealed that an increased HDL‐C did not reduce the risk of CVD or mortality,^21^ the research focus on HDL‐C is moving toward their function in cholesterol efflux capacity.^8^ Additionally, the American Association of Clinical Endocrinologists recommends that lipid values should be monitored every 6 weeks until target levels are achieved.^22^

Glucometers are commonly used by patients with diabetes to routinely check their blood glucose levels despite the controversy over the accuracy of some devices.^23^, ^24^ In comparison, POC devices for lipid panel testing have not yet been widely used. Considering that patients taking therapeutic drugs for dyslipidemia have to visit a hospital regularly to undergo lipid testing, the ability to perform lipid and glucose tests together would be convenient. The newly developed GCare Lipid Analyzer could prove beneficial to patients because it works similarly to a conventional glucometer.

Although the concept of performing multiple tests using a single device is not new, there may be many potential barriers obstructing the successful development, validation, and implementation of novel POC analyzers.^25^ Nonetheless, the verification of the analytical performance of newly developed POC devices is important. Considering that measurements of lipid profiles and glucose concentrations are essential for the risk assessment of CVD and monitoring diabetes, providing accurate and reliable results is the most essential and basic requirement of such devices. In this study, the assured quality of POC devices is ensured through effective operator training and compliance with the manufacturer's technical guidelines; therefore, sufficient information was provided to the technicians and users.^26^

The total error, which reflects both bias and impression in the NCEP criteria, was met by the GCare Lipid Analyzer using venous blood. The overall analytical imprecision (%) of TC and HDL‐C was slightly higher than the CV recommended by the NCEP and CDC, although the mean bias (%) was lower than that of the values in the guidelines. The NCEP guidelines do not differentiate between the measurements obtained in the laboratory and those acquired using the alternative setup of a desktop analyzer.^27^ Therefore, these criteria can be challenging when applied to POC devices. Nevertheless, our study revealed an acceptable total error for the GCare Lipid Analyzer.

The correlation study showed acceptable agreement between the reference values obtained using the Toshiba TBA 2000FR analyzer by both technicians and users. The mean bias of TG and HDL‐C was slightly higher for the capillary blood measurements than for the venous blood measurements, although the difference was not significant. A comparative study of the values obtained by users and technicians and sample type (capillary vs. venous blood) revealed a good correlation and acceptable bias.

Although some misclassified cases were present in the clinical agreement categorization, none were classified outside of one category. Some of the TC values were misclassified as ≥200 and <240 mg/dl because the POC value was 200 mg/dl, the exact cut‐off value, so it is considered that the misclassification rate was overestimated. In TG, one capillary blood case with a reference value of 200 mg/dl was misclassified as lower category of ≥150 to <200 mg/dl, with a POC value of 189 mg/dl (indicated in bold letters in Table 3). The threshold for treating dyslipidemia is a TG level of 200 mg/dl, as mentioned above. This underestimation can be problematic for users performing capillary blood measurements at home. However, since low HLD‐C levels (<40 mg/dl) are also included in the criteria for determining the treatment policy, the possibility of simultaneous underestimation of TG and false HDL‐C seems low.

The GCare Lipid Analyzer using the GCare Glucose Test Strip was evaluated according to ISO 15197:2013 guidelines. The two criteria stated in the minimum system accuracy performance criteria were met by the capillary and venous blood samples. In addition, the user performance evaluation revealed acceptable results for glucose concentrations of <100 mg/dl and ≥100 mg/dl. There was a minimal effect due to interfering substances that exceeded the performance criteria in the interference analysis.

Our study has some limitations. First, the exact fasting hours of the test subjects were not clear. The NCEP recommendations state that TG, HDL‐C, and LDL‐C measurements should preferably be taken from samples collected after a 12‐h fast.^28^ The minimal fasting hours were achieved because the test subjects visited the hospital for lipid testing. However, in our study, it was difficult to determine whether strict 12‐h fasting was performed; this factor might have affected the measurements. Second, the appropriate glucose concentration distribution and the acceptable samples that can be modified in the very low and very high glucose concentrations for evaluation are given in the ISO guidelines. In our study, none of the blood samples in bins 3 to 5 were altered according to the guidelines. However, in bins 2 and 6, a larger number of spiked samples than the number suggested in the ISO guidelines were used because of the shortage of available samples. Finally, when the GCare Lipid Analyzer value and the reference value were compared, an overall negative mean bias was detected (capillary blood: −7.8 mg/dl; venous blood: −0.4 mg/dl). The bias was greater when capillary blood was used and further increased as the blood glucose concentration increased. Although these biases were within the acceptable range suggested by the ISO 15197:2013 guidelines, underestimating the blood glucose value may put patients at risk especially in those with hyperglycemia. Patients who routinely measure their blood glucose using capillary blood should be aware of the possibility of bias.

In this study, the GCare Lipid Analyzer demonstrated good clinical agreement with the reference values for TC, TG, and HDL‐C using capillary and venous blood. The analytical performance based on the NCEP criteria was also acceptable. The GCare Glucose Test Strip meets the requirements for system accuracy indicated in the ISO 15197:2013 guidelines. The acceptable results of the user performance evaluation suggest that the device is reliable when used by non‐experts. Thus, the GCare Lipid Analyzer is expected to benefit patients by facilitating the convenient monitoring of blood glucose and lipid levels.

## CONFLICT OF INTEREST

The author(s) declare no potential conflicts of interest with respect to the research, authorship, and/or publication of this article.

## Supporting information

Supplementary Material

## Data Availability

The data that support the findings of this study are available from the corresponding author upon reasonable request.
