# Supplementary material for: Simultaneous point‐of‐care testing of blood lipid profile and glucose: Performance evaluation of the GCare Lipid Analyzer
Source: J Clin Lab Anal. 2021 Oct 25;35(12):e24055. doi: 10.1002/jcla.24055 (PMC8649338; doi:10.1002/jcla.24055)
Supplement: Supplementary file 1 — Supplementary Material [file JCLA-35-e24055-s001.docx]

**Supplementary Table 1.** Precision data of the GCare Lipid Analyzer.

| Item | Level  (mg/dL) | Reference value (mg/dL) | Lot 1 | | | Lot 2 | | | Lot 3 | | |  |  |  |
| --- | --- | --- | --- | --- | --- | --- | --- | --- | --- | --- | --- | --- | --- | --- |
|  |  |  | Mean (mg/dL) | SD (mg/dL) | CV (%) | Mean (mg/dL) | SD (mg/dL) | CV (%) | Mean (mg/dL) | SD (mg/dL) | CV (%) | Grand  mean (mg/dL) | Pooled  SD (mg/dL) | Pooled  CV (%) |
| TC | Low  (130-160) | 142 | 140.9 | 4.9 | 3.5 | 139.3 | 6.7 | 4.8 | 137.1 | 5.9 | 4.3 |  |  |  |
|  | Middle  (180-210) | 208.5 | 195.7 | 6.1 | 3.1 | 205.8 | 9.3 | 4.5 | 202.9 | 8.5 | 4.2 |  |  |  |
|  | High  (220-250) | 237.5 | 242.3 | 6.3 | 2.6 | 246.3 | 10.0 | 4.1 | 251.8 | 7.3 | 2.9 |  |  |  |
| TG | Low  (90-120) | 114.5 | 121.9 | 5.3 | 4.3 | 122.5 | 5.9 | 4.8 | 120.2 | 5.0 | 4.2 |  |  |  |
|  | Middle  (140-180) | 165.5 | 172.8 | 6.1 | 3.5 | 171.0 | 6.3 | 3.7 | 176.7 | 5.8 | 3.3 |  |  |  |
|  | High  (200-250) | 225.5 | 214.7 | 4.1 | 1.9 | 217.0 | 7.1 | 3.3 | 214.9 | 6.8 | 3.2 |  |  |  |
| HDL-C | Low  (30-40) | 34 | 37.0 | 1.7 | 4.6 | 37.8 | 0.8 | 2.1 | 38.5 | 2.3 | 5.9 |  |  |  |
|  | Middle  (45-55) | 52 | 53.7 | 2.7 | 5.0 | 54.9 | 2.2 | 4.1 | 53.3 | 2.2 | 4.2 |  |  |  |
|  | High  (60-75) | 73.5 | 79.2 | 3.2 | 4.1 | 80.5 | 2.8 | 3.5 | 80.9 | 2.5 | 3.1 |  |  |  |
| Glucose | level 1 | 47.3 | 45.3 | 3.0 | - | 44.8 | 2.9 | - | 44.9 | 2.7 | - | 45.0 | 2.9 |  |
|  | level 2 | 74.1 | 77.5 | 3.4 | - | 75.6 | 3.0 | - | 75.6 | 2.5 | - | 76.2 | 3.0 |  |
|  | level 3 | 127.5 | 129.9 | 3.8 | 2.9 | 129.1 | 3.2 | 2.5 | 131.4 | 4.5 | 3.4 | 130.1 | 3.9 | 3.0 |
|  | level 4 | 209.2 | 212.7 | 6.1 | 2.9 | 210.6 | 5.5 | 2.6 | 212.1 | 5.0 | 2.3 | 211.8 | 5.5 | 2.6 |
|  | level 5 | 353.8 | 323.3 | 5.9 | 1.8 | 323.6 | 6.8 | 2.1 | 319.0 | 5.2 | 1.6 | 322.0 | 6.0 | 1.9 |

Acceptance criteria of CV: TC≤3 %, TG≤5 %, HDL-C≤4 %.

CV, coefficient of variation; HDL-C, high-density lipoprotein cholesterol; SD, standard deviation; TC, total cholesterol; TG, triglycerides

**Supplementary Table 2.** Passing–Bablok regression analysis of the values obtained by the GCare Lipid Analyzer from capillary and venous blood versus the values obtained using the Toshiba TBA2000FR analyzer as a reference.

|  | Capillary |  |  | Venous |  |  |
| --- | --- | --- | --- | --- | --- | --- |
|  | TC | TG | HDL-C | TC | TG | HDL-C |
| Number | 134 | 124 | 136 | 132 | 126 | 136 |
| Range | 103 to 292.5 | 51 to 483.5 | 26 to 95 | 106 to 306.5 | 50 to 483 | 27 to 90 |
| Intercept | -5.338  (-14.48 to 1.631) | 2.500  (-1.789 to 5.723) | -2.200  (-5.312 to 1.000) | -10.17  (-19.32 to -0.750) | 3.000  (-0.180 to 5.705) | 0.000  (-1.632 to 2.819) |
| Slope | 1.038  (0.9970 to 1.087) | 1.000  (0.976 to 1.029) | 1.066  (1.000 to 1.125) | 1.050  (1.000 to 1.103) | 1.000  (0.977 to 1.029) | 1.000  (0.953 to 1.029) |
| Correlation coefficient (R) | 0.965  (0.951 to 0.975) | 0.968  (0.955 to 0.978) | 0.943  (0.920 to 0.959) | 0.969  (0.957 to 0.978) | 0.990  (0.985 to 0.993) | 0.956  (0.938 to 0.968) |

HDL-C, high-density lipoprotein cholesterol; TC, total cholesterol; TG, triglycerides

**Supplementary Table 3.** Evaluation of hematocrit (Hct) effect at five Hct volumes and three glucose concentration intervals. The difference between the average measured glucose level at each Hct in interval 1 (<100 mg/dL) is expressed as mg/dL, whereas the differences in intervals 2 and 3 (≥100 mg/dL) are expressed as a percentage (%) according to ISO 15197:2013 guidelines.

| Glucose interval  (mg/dL) | Glucose YSI  (mg/dL) | Lot 1 | | | | | Lot 2 | | | | | Lot 3 | | | | |
| --- | --- | --- | --- | --- | --- | --- | --- | --- | --- | --- | --- | --- | --- | --- | --- | --- |
|  | Hct (%) | 21% | 31.8% | 40.5% | 50.4% | 61.5% | 21% | 31.8% | 40.5% | 50.4% | 61.5% | 21% | 31.8% | 40.5% | 50.4% | 61.5% |
| 1  (30-50) | 46 | -5.8 | -6.5 | 0.0 | 5.9 | 5.4 | -7.3 | -5.3 | 0.0 | 4.4 | 2.5 | -8.1 | -5.4 | 0.0 | 6.7 | 5.0 |
| 2  (96-144) | 115 | 6.3 | -4.1 | 0.0 | -5.1 | -1.7 | 6.8 | -8.6 | 0.0 | -5.7 | -1.1 | 9.5 | -4.8 | 0.0 | -2.6 | -2.0 |
| 3  (280-420) | 308 | 7.1 | 1.4 | 0.0 | -4.0 | -6.2 | 2.7 | -2.7 | 0.0 | -4.4 | -8.4 | 7.2 | 0.2 | 0.0 | -2.7 | -6.1 |

**Supplementary Table 4.** Summary of interference testing for 24 substances specifying the concentration with no significant interference

|  |  | Glucose concentration (mg/dL) | |
| --- | --- | --- | --- |
| Substance | Concentration | <100 | ≥100 |
|  |  | Bias (mg/dL) | Bias (%) |
| Acetaminophen | 20 mg/dL | 1.2 | 1.6 |
| Ascorbate | 3 mg/dL | 6.1 | 2.1 |
| Bilirubin | 50 mg/dL | -3.9 | 1.5 |
| Cholesterol | 500 mg/dL | -2.1 | 1.6 |
| Creatinine | 10 mg/dL | -0.9 | 0.5 |
| EDTA | 200 mg/dL | 1.7 | 2.4 |
| Galactose | 15 mg/dL | 0.4 | 0 |
| Hemoglobin | 20000 mg/dL | -8.2 | -8.3 |
| Heparin | 500 U/dL | 0.4 | 0.2 |
| Ibuprofen | 50 mg/dL | 0.8 | 1.9 |
| Icodextrin | 1095 mg/dL | -3.8 | -1.6 |
| Pralidoxime iodide | 25 mg/dL | -0.4 | -3.3 |
| L-DOPA | 0.5 mg/dL | 8.2 | 3.3 |
| Maltose | 10000 mg/dL | -1.8 | -6.6 |
| Salicylate | 60 mg/dL | 0.8 | 2 |
| Tolbutamide | 100 mg/dL | -1.8 | 1.2 |
| Triglycerides | 1500 mg/dL | 2.1 | 4.5 |
| Xylose | 200 mg/dL | 6.1 | 2.8 |
| Dopamine^a^ | 1.2 mg/dL | concentrations determined from dose-response evaluation | |
| Gentisic acid^a^ | 5 mg/dL |  |  |
| Glutathione^a^ | 4.5 mg/dL |  |  |
| Urate^a^ | 8.5 mg/dL |  |  |
| Methyldopa^a^ | 4.5 mg/dL |  |  |
| Tolazamide^a^ | 26 mg/dL |  |  |

^a^Substances for which dose-response evaluation was performed.

EDTA, ethylenediaminetetraacetic acid; l-DOPA, levodopa

**Supplementary Table 5.** Blood glucose concentrations of samples in the system accuracy evaluation

| Interval | Glucose concentration (mg/dL): ISO | Percentage of sample (%): ISO | Sample distribution of capillary blood  (modified sample) | Sample distribution of venous blood  (modified sample) |
| --- | --- | --- | --- | --- |
| 1 | ≤50 | 5 | (5) | (5) |
| 2 | >50–80 | 15 | 9 (6) | 14 (1) |
| 3 | >81–120 | 20 | 20 | 20 |
| 4 | >121–200 | 30 | 30 | 30 |
| 5 | >201–300 | 15 | 15 | 15 |
| 6 | >301–400 | 10 | 6 (4) | 5 (5) |
| 7 | ≥400 | 5 | 2 (3) | 2 (3) |
| Total |  |  | 82 (18) | 86 (14) |

ISO, International Organization for Standardization.

Modified samples are indicated by numbers in parentheses.

**
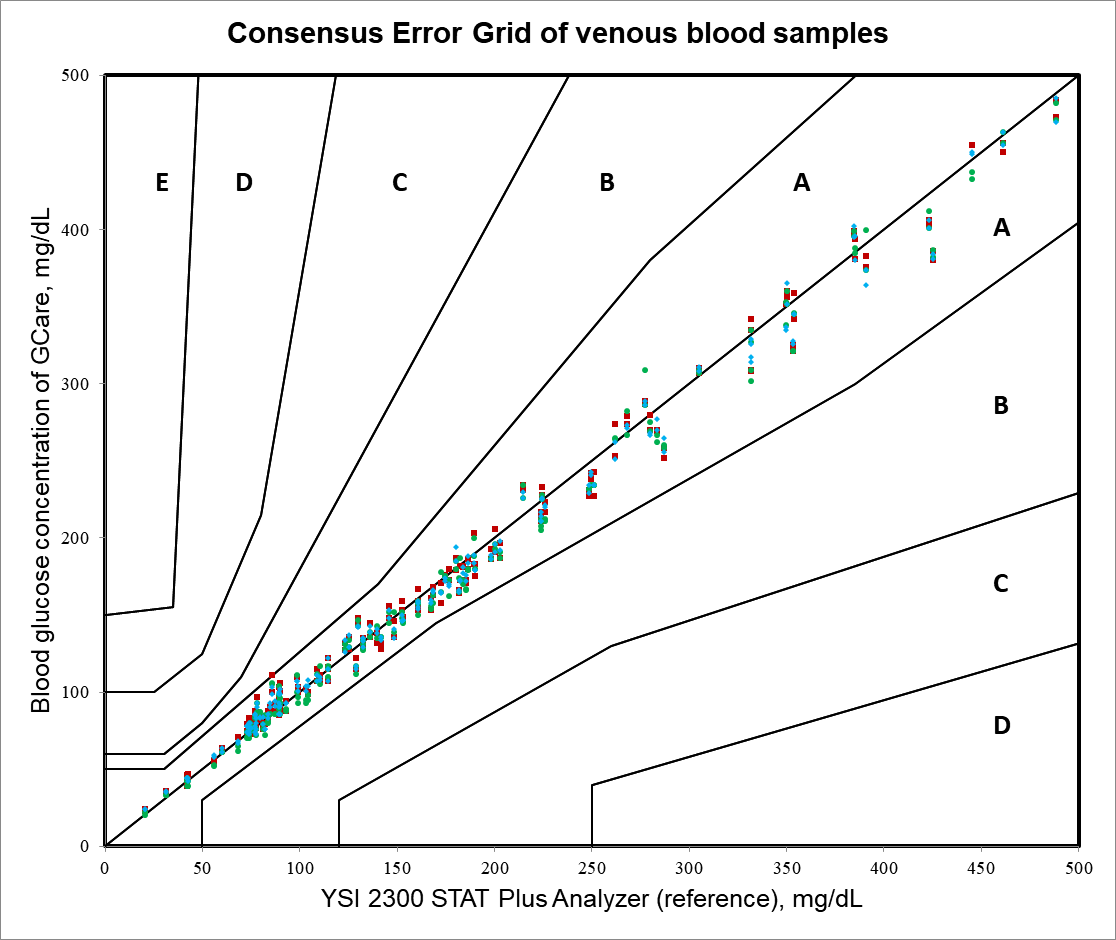
**

**(A)**

**
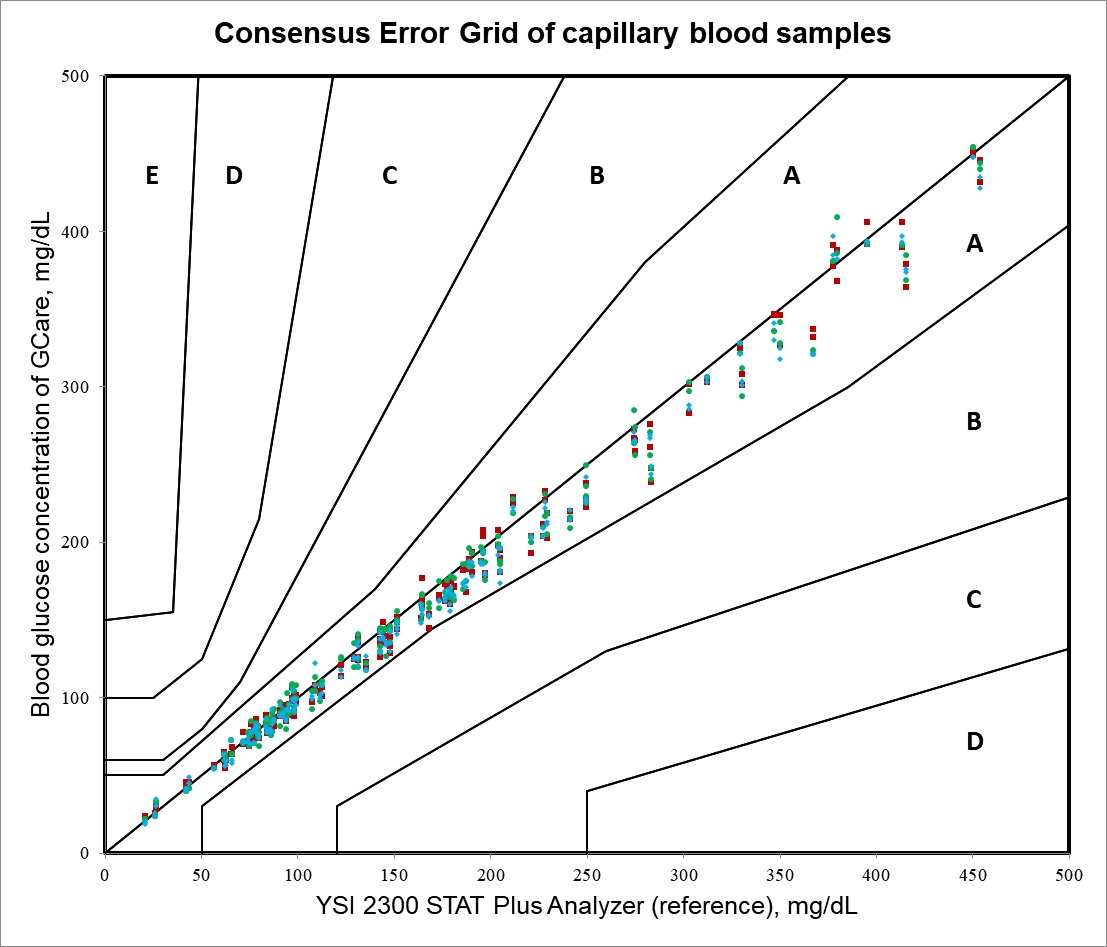
**

**(B)**

**
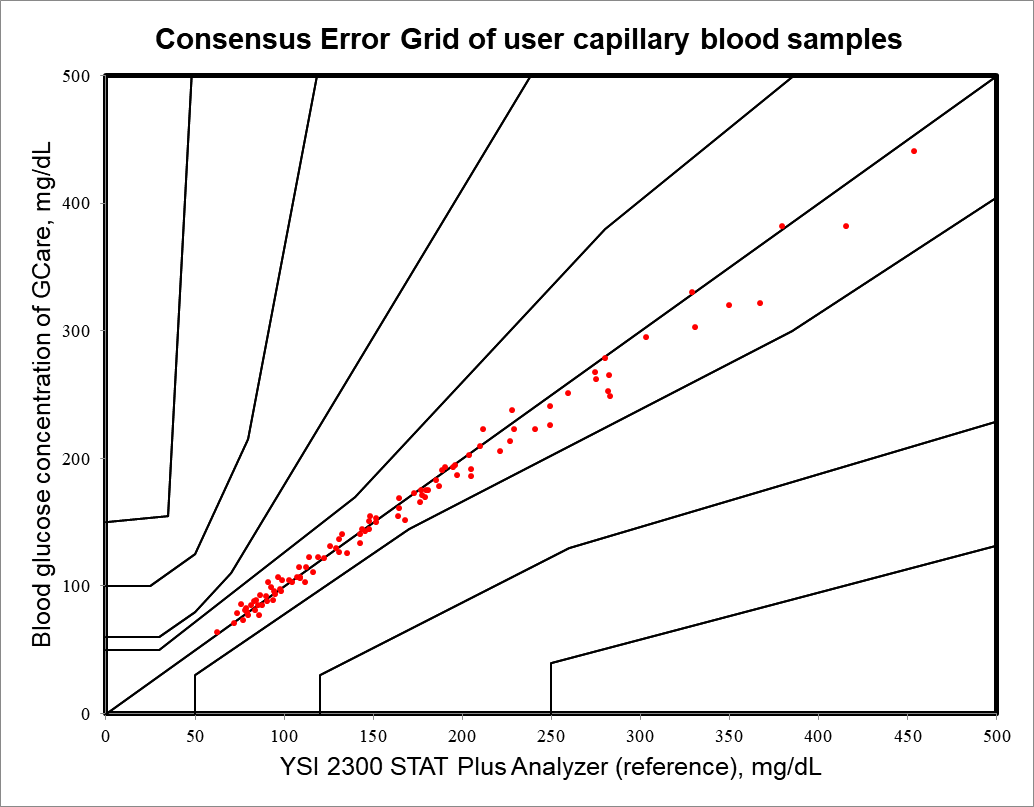
**

**(C)**

**Supplementary Figure 1.** CEG of the measured glucose levels using a GCare Lipid Analyzer using a GCare Glucose Test Strip obtained from three different lots using (A) venous and (B) capillary blood samples. (C) CEG of user capillary blood glucose results performed from one lot.

Zone A: no effect on clinical action

Zone B: altered clinical action (little or no effect on clinical outcome)

Zone C: altered clinical action (likely to affect clinical outcome)

Zone D: altered clinical action (could have significant medical risk)

Zone E: altered clinical action (could have dangerous consequences)

CEG, consensus error grid

**Supplementary Table 6.** Characteristics of test subjects with diabetes for user performance evaluation

| Characteristic | All subjects (*n* = 100) |
| --- | --- |
| Sex, *n* |  |
| Male | 49 |
| Female | 51 |
| Age, *n* |  |
| 20 – 40 | 18 |
| 41 – 50 | 14 |
| 51 – 60 | 24 |
| ≥ 61 | 44 |
| Education level, *n* |  |
| Elementary school | 6 |
| Middle school | 22 |
| High school | 35 |
| Higher than college | 37 |
| Glucose concentration, *n* |  |
| > 50–80 mg/dL | 9 |
| > 80–120 mg/dL | 30 |
| > 120–200 mg/dL | 34 |
| > 200–300 mg/dL | 19 |
| > 300–400 mg/dL | 6 |
| ≥ 400 mg/dL | 2 |
